# An Unusual Presentation of Antiphospholipid Syndrome With Seizures and Sagittal Sinus Thrombosis: A Case Report

**DOI:** 10.7759/cureus.95977

**Published:** 2025-11-03

**Authors:** Basma Alleelwa, Mohammed Haitham Faeq Faeq, Aliyu O Olaniyi

**Affiliations:** 1 Acute Medicine, Stepping Hill Hospital, Stockport, GBR; 2 Critical Care Medicine, King's College Hospitals NHS Foundation Trust, London, GBR; 3 Geriatrics, Stepping Hill Hospital, Stockport, GBR

**Keywords:** antiphospholipid antibody syndrome (aps), autoimmune stroke mimic, cerebral venous sinus thrombosis (cvst), haemorrhagic infarction with seizures, lupus anticoagulant, superior sagittal sinus thrombosis, unprovoked thrombosis

## Abstract

Cerebral venous sinus thrombosis (CVST) is a rare but potentially life-threatening form of stroke. It may present with nonspecific neurological symptoms, including headache, seizures, or focal deficits. One of the rare but important underlying causes is antiphospholipid syndrome (APS), an autoimmune prothrombotic condition associated with persistent antiphospholipid antibodies: lupus anticoagulant (LA), anticardiolipin (aCL), or anti-β2 glycoprotein I (anti-β2GPI). APS-related CVST is typically seen in young women, often with systemic autoimmune disease such as systemic lupus erythematosus.

We present a rare and atypical case of a 55-year-old man with no prior autoimmune history who developed new-onset seizures and right-sided weakness while visiting the UK. Imaging revealed a haemorrhagic infarct in the left frontal lobe secondary to superior sagittal sinus thrombosis. Initial thrombophilia screening identified an isolated positive lupus anticoagulant, with confirmatory repeat testing at 12 weeks fulfilling the revised Sapporo criteria for APS. The patient was managed with anticoagulation, antiepileptics, and specialist follow-up.

This case is unusual due to several factors: the patient’s demographic (middle-aged male), the absence of systemic autoimmune disease, presentation with seizures and intracerebral haemorrhage, and the isolated lupus anticoagulant positivity without triple antibody presence.

It highlights the importance of considering APS in unprovoked CVST even in the absence of classic risk factors. Early diagnosis, appropriate imaging, and timely anticoagulation can improve outcomes. This case reinforces the need for autoimmune and thrombophilia screening in atypical stroke presentations.

## Introduction

Antiphospholipid syndrome (APS) is an autoimmune disorder characterised by a persistent prothrombotic state caused by circulating antiphospholipid antibodies: lupus anticoagulant (LA), anticardiolipin (aCL), and anti‑β₂‑glycoprotein I (anti‑β₂GPI), in association with clinical evidence of vascular thrombosis or pregnancy morbidity [1]. APS may occur as a primary disease or secondary to systemic autoimmune conditions, most commonly systemic lupus erythematosus. Its typical clinical manifestations include venous or arterial thrombosis, recurrent pregnancy loss, and thrombocytopenia [2].

Although APS most often presents with deep vein thrombosis or pulmonary embolism, neurological complications are increasingly recognised. These include ischaemic stroke, transient ischaemic attacks, cognitive impairment, and, more rarely, cerebral venous sinus thrombosis (CVST) [2,3]. CVST results from thrombosis of the dural venous sinuses, leading to impaired venous drainage, venous congestion, infarction, and occasionally intracerebral or subarachnoid haemorrhage [3-5]. CVST typically presents with variable and nonspecific symptoms such as headache, seizures, focal deficits, or altered consciousness, which often delay diagnosis [5,6].

The coexistence of APS and CVST is uncommon and diagnostically challenging, particularly when the presentation occurs in patients without classical risk factors or systemic autoimmune disease. Isolated lupus anticoagulant positivity, as seen in some cases, has been identified as a clinically relevant yet often overlooked thrombotic risk [4,7].

This report describes a rare presentation of primary APS manifesting as CVST with haemorrhagic infarction and seizures in a previously healthy middle‑aged man as the first presentation. The case underscores the need for a high index of suspicion for APS in patients presenting with unprovoked CVST and highlights the diagnostic and therapeutic implications of identifying an autoimmune aetiology early in management [8]. CVST accounts for approximately 0.5-1% of all strokes [9]. 

## Case presentation

A 55-year-old male tourist from Hong Kong presented to the emergency department with a sudden onset of generalised tonic-clonic seizure lasting approximately two minutes, witnessed by his wife. He was postictal for several minutes and subsequently developed right-sided hemiparesis. There was no prior history of seizures, thrombotic events, or autoimmune disease. On examination, he was alert (GCS 15), oriented, and haemodynamically stable. Neurological assessment revealed right-sided upper and lower limb weakness (MRC grade 3/5), with mild right-sided sensory impairment. Cranial nerve examination was normal, and there were no signs of meningism or raised intracranial pressure.

**Figure 1:**
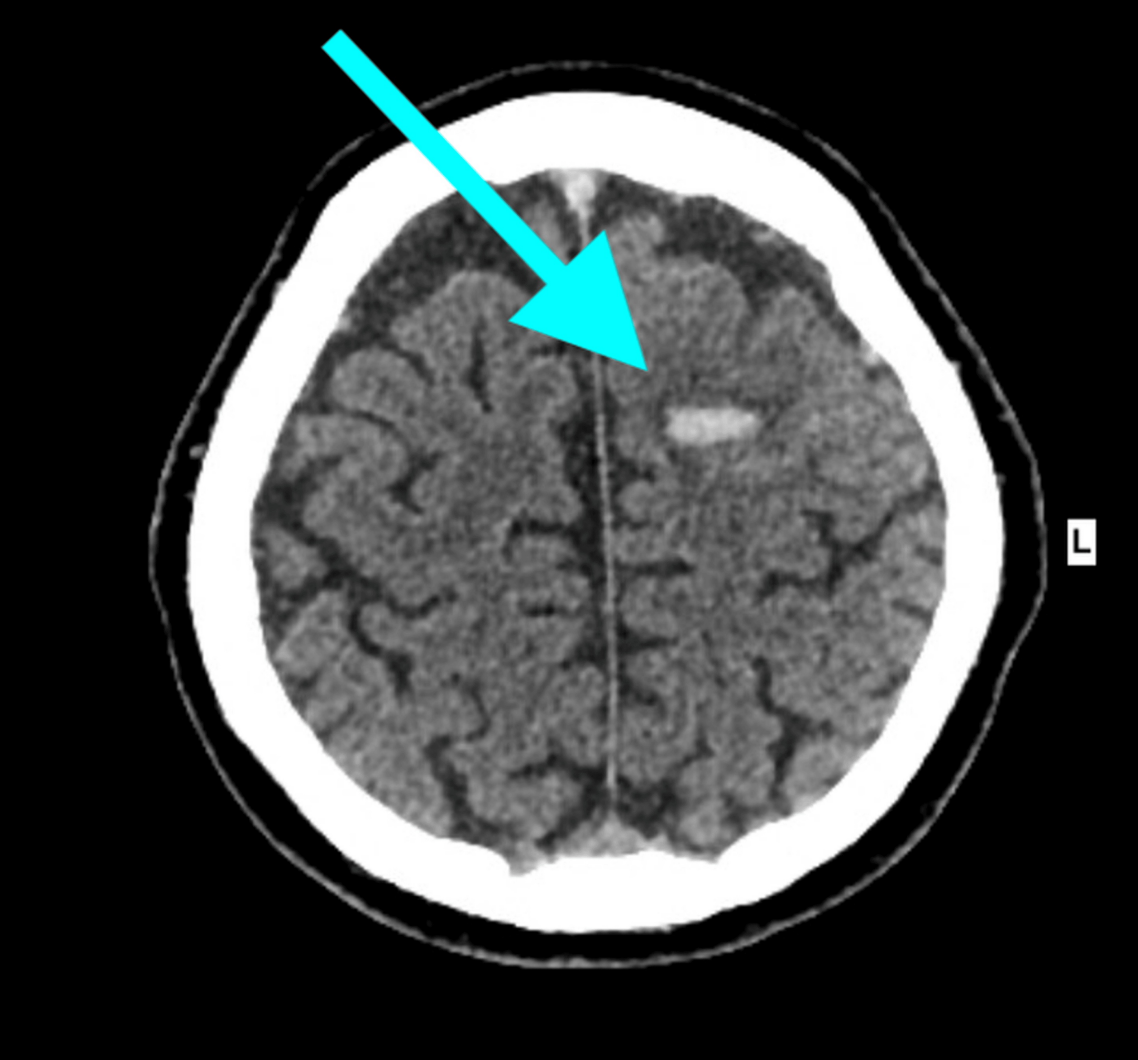
Non-contrast CT head shows a small acute intracerebral haemorrhage in the superior portion of the left frontal lobe (blue arrow)

Initial non-contrast CT head revealed a small acute left frontal lobe haemorrhage (Figure 1).

**Figure 2:**
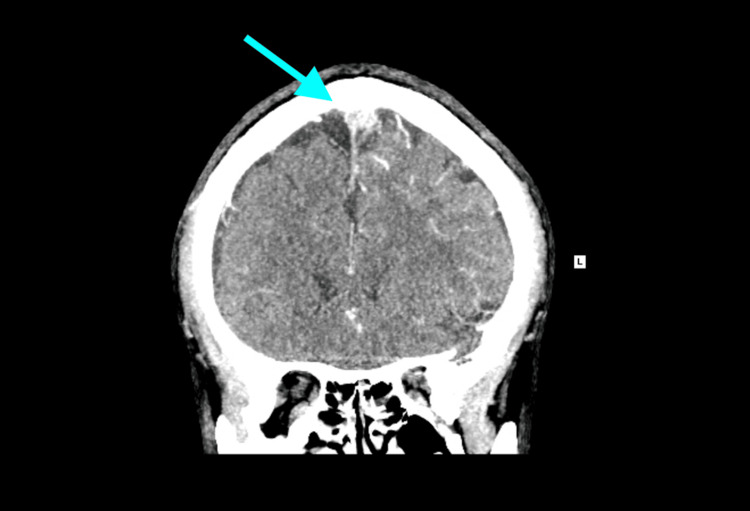
CT venography shows superior sagittal sinus thrombosis (blue arrow)

CT venography demonstrated thrombosis of the superior sagittal sinus and the left anterior cortical vein, consistent with haemorrhagic venous infarction (Figure 2).

**Figure 3:**
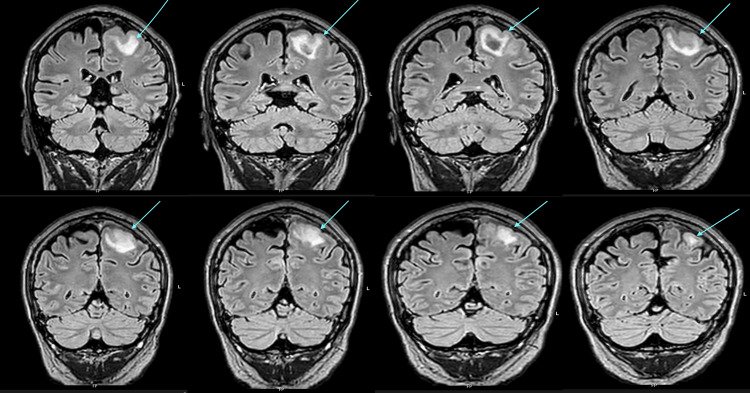
MRI brain (FLAIR (Fluid-Attenuated Inversion Recovery) sequences) confirms a left frontal parenchymal haemorrhage (blue arrow), with minor diffusion restriction and perifocal oedema.

MRI confirmed these findings and excluded mass lesions or arteriovenous malformations (Figure 3).

Laboratory investigations showed normal full blood count, renal and liver function, CRP, and coagulation profile (INR 1.0). Lipid profile was significantly elevated (cholesterol 8.7 mmol/L, LDL 6.4 mmol/L), prompting the initiation of statin therapy. Thrombophilia screening revealed a positive lupus anticoagulant, with negative anticardiolipin and anti-β2 glycoprotein I antibodies (Table 1).

**Table 1:** Laboratory tests DRVVT: Dilute Russell's Viper Venom Time; PGM: Prothrombin Gene Mutation; HbA1c: Hemoglobin A1c; Protein S Free Ag: Protein S Free Antigen; Serum ALT: Serum Alanine Aminotransferase; Serum GGT: Serum Gamma-Glutamyl Transferase; Serum LDL Cholesterol: Serum Low-Density Lipoprotein Cholesterol; HDL: High-Density Lipoprotein; PT: Prothrombin Time; INR: International Normalized Ratio; APTT: Activated Partial Thromboplastin Time.

Test	Result	Reference
Factor V Leiden	Not detected	-
Lupus Screen	Lupus Anticoagulant detected	-
DRVVT Screen Ratio	1.30	≤ 1.15
DRVVT Confirm	0.88	≤ 1.20
DRVVT Normalised ratio	1.47	≤ 1.20
DRVVT Interpretation	Positive	-
Antithrombin III	101 IU/dL	(83 - 128) IU/dL
Protein C Functional	130.0 IU/dL	(70.0 - 140.0) IU/dL
Protein S Free Ag	77.3 IU/dL	(74.0 - 146.0) IU/dL
PGM G20210A	Not detected	-
White Cell Count	4.7 10*9/L	(3.7 - 11.0) 10*9/L
Red Cell Count	5.35 10*12/L	(4.50 - 6.50) 10*12/L
Haemoglobin	156 g/L	(130 - 180) g/L
Haematocrit (PCV)	0.47 L/L	(0.40 - 0.54) L/L
Mean Cell Volume	87.3 fL	(76.0 - 100.0) fL
Mean Cell Hgb	29.2 pg	(27.0 - 32.0) pg
Mean Cell Hgb Conc	335 g/L	(320 - 365) g/L
Red Cell Dist Width	14 %	(11.5 - 14.5) %
Platelet Count	170 10*9/L	(150 - 450) 10*9/L
Neutrophils	3.1 10*9/L	(1.7 - 7.5) 10*9/L
Lymphocytes	1.1 10*9/L	(1.0 - 4.5) 10*9/L
Monocytes	0.4 10*9/L	(0.2 - 1.1) 10*9/L
Eosinophils	0.1 10*9/L	(0.0 - 0.6) 10*9/L
Basophils	0.0 10*9/L	(0.0-0.1) 10*9/L
Cardiolipin G	2.00 U	0.00-19.90 U
Cardiolipin M	< 1.50 U	0.00-19.90 U
HbA1c	44 mmol/mol	(30-41) mmol/mol
Serum Total Bilirubin	12umol/L	(1 - 21) umol/L
Serum ALT	26U/L	(0 - 45) U/L
Alkaline phosphatase	57U/L	(20 - 130) U/L
Serum GGT	22U/L	(12 - 64) U/L
Serum Total Protein	76g/L	(60 - 80) g/L
Serum Albumin	45g/L	(38 - 51) g/L
Serum Globulins	31g/L	(18 - 35) g/L
Serum Cholesterol	8.7mmol/L	< 5.2 mmol/L
Serum HDL Cholesterol	1.6 mmol/L	(1.0–1.5) mmol/L
Serum Triglycerides	1.6 mmol/L	< 1.7 mmol/L
Serum LDL Cholesterol	6.4 mmol/L	< 2.6 mmol/L
Cholesterol HDL ratio	5.44 mmol/L	< 5.0 mmol/L
Non-HDL cholesterol	7.1 mmol/L	< 3.4 mmol/L
PT	11.2 seconds	11.0-13.5 seconds
APTT	30.3 seconds	25-35 seconds
APTT Ratio	0.90	0.8-1.2
INR	1.0	0.8-1.2

Malignancy screening with CT chest, abdomen, and pelvis was unremarkable. The autoimmune panel for APS was negative apart from a persistently positive lupus anticoagulant. Autoimmune screening (e.g., ANA, dsDNA) was not performed, as there were no clinical features of systemic autoimmune disease. In the absence of such features, the presentation was attributed to primary APS. He was commenced on therapeutic enoxaparin (1 mg/kg BD) despite the presence of haemorrhage, in line with CVST management guidelines [7]. Levetiracetam was initiated and titrated to control seizures. Neurosurgical opinion favoured conservative management. After seven days, he was transitioned to oral anticoagulation with dabigatran 150mg twice daily. During his admission, the patient showed progressive neurological improvement, with near-complete recovery of right-sided motor strength and resolution of sensory deficits by day ten. No further seizures were reported following initiation of levetiracetam. He was mobilised with physiotherapy support and discharged home on dabigatran, levetiracetam, and atorvastatin.

On follow-up at six weeks in the neurology clinic, he remained seizure-free with full neurological recovery. A CT venogram cerebral was arranged for six months by the neurology team to assess superior sagittal sinus recanalisation. A multidisciplinary decision was made to continue long-term anticoagulation in view of his positive lupus anticoagulant, with haematology follow-up arranged. He was also reviewed by the smoking cessation team as part of his follow-up and was advised on lifestyle modification and strict adherence to anticoagulation, with ongoing review in stroke and haematology clinics.

At 12-week follow-up, repeat antiphospholipid antibody testing confirmed persistent positivity for lupus anticoagulant, fulfilling the revised Sapporo/Sydney criteria for APS. The DRVVT screen ratio was 1.32 (ref ≤1.15), APTT ratio 0.90 (ref ≤1.20), and normalised ratio 1.45 (ref ≤1.20).

## Discussion

This case illustrates an atypical presentation of APS with CVST and haemorrhagic infarction. CVST represents approximately 0.5% of all strokes and is more commonly associated with prothrombotic conditions, including APS [2,3]. Seizures occur in nearly one-third of CVST cases, particularly with superior sagittal sinus involvement [7].

APS is defined by the presence of clinical thrombosis or pregnancy morbidity in conjunction with persistent antiphospholipid antibodies, including lupus anticoagulant, anticardiolipin antibodies, and anti-β2 glycoprotein I antibodies [2]. The most frequent clinical presentations of APS include deep vein thrombosis of the lower limbs, pulmonary embolism, and recurrent miscarriages [5]. Neurological manifestations are less common but can include ischaemic stroke, transient ischaemic attacks, and rarely, CVST [2,6].

This case is considered unusual because CVST is a rare neurological manifestation of APS, and even more so when associated with an intracerebral haemorrhage and seizures in a previously healthy, middle-aged male without traditional thrombotic risk factors [7]. Most CVST cases in APS occur in younger women and are often associated with other systemic autoimmune conditions, such as systemic lupus erythematosus [6]. In contrast, our patient was older, male, and had no known autoimmune background or provoking factors, making the presentation highly atypical. This case contributes to the literature by reinforcing the importance of recognising APS as an underlying cause of CVST even in patients who fall outside typical demographic profiles, thereby supporting broader diagnostic consideration in atypical stroke presentations.

Despite radiological evidence of intracranial haemorrhage, anticoagulation remains the cornerstone of CVST treatment, even in the context of APS [4,7]. Low-molecular-weight heparin is typically used initially, followed by oral anticoagulation. Although direct oral anticoagulants like dabigatran are not currently first-line in APS, they may be considered in select patients with low-risk profiles and no triple antibody positivity [8].

Long-term anticoagulation is usually required in confirmed APS due to a high risk of recurrence [2,5]. Seizure management with antiepileptic drugs is warranted in patients with symptomatic CVST, though long-term prophylaxis remains controversial [7].

## Conclusions

This case highlights the need to consider APS in patients presenting with unprovoked CVST, particularly when associated with seizures and haemorrhagic infarction. The presentation in a previously healthy middle-aged man without known risk factors underscores the importance of maintaining a broad differential diagnosis. Early neuroimaging, autoimmune screening, and prompt anticoagulation are essential for optimal outcomes. Early recognition of APS in atypical stroke presentations is critical to avoid delays in treatment and reduce the risk of recurrence. Long-term management includes patient education about recurrence risk and treatment adherence. Further research is needed to establish diagnostic pathways and optimise treatment strategies in atypical presentations of APS with CVST.
